# Nest Making and Oxytocin Comparably Promote Wound Healing in Isolation Reared Rats

**DOI:** 10.1371/journal.pone.0005523

**Published:** 2009-05-13

**Authors:** Antonia Vitalo, Jonathan Fricchione, Monica Casali, Yevgeny Berdichevsky, Elizabeth A. Hoge, Scott L. Rauch, Francois Berthiaume, Martin L. Yarmush, Herbert Benson, Gregory L. Fricchione, John B. Levine

**Affiliations:** 1 Department of Psychiatry, Massachusetts General Hospital, Boston, Massachusetts, United States of America; 2 Department of Surgery, Massachusetts General Hospital, Boston, Massachusetts, United States of America; 3 Department of Medicine Services, Massachusetts General Hospital, Boston, Massachusetts, United States of America; 4 Benson Henry Institute for Mind Body Medicine, Massachusetts General Hospital, Boston, Massachusetts, United States of America; 5 Center for Engineering and Medicine, Massachusetts General Hospital, Boston, Massachusetts, United States of America; 6 Shriners Burns Hospital, Boston, Massachusetts, United States of America; 7 Department of Psychiatry, Harvard Medical School, Boston, Massachusetts, United States of America; 8 Department of Surgery, Harvard Medical School, Boston, Massachusetts, United States of America; 9 Department of Medicine, Harvard Medical School, Boston, Massachusetts, United States of America; 10 McLean Hospital, Belmont, Massachusetts, United States of America; Chiba University Center for Forensic Mental Health, Japan

## Abstract

**Background:**

Environmental enrichment (EE) fosters attachment behavior through its effect on brain oxytocin levels in the hippocampus and other brain regions, which in turn modulate the hypothalamic-pituitary axis (HPA). Social isolation and other stressors negatively impact physical healing through their effect on the HPA. Therefore, we reasoned that: 1) provision of a rat EE (nest building with Nestlets®) would improve wound healing in rats undergoing stress due to isolation rearing and 2) that oxytocin would have a similar beneficial effect on wound healing.

**Methodology/Principal Findings:**

In the first two experiments, we provided isolation reared rats with either EE or oxytocin and compared their wound healing to group reared rats and isolation reared rats that did not receive Nestlets or oxytocin. In the third experiment, we examined the effect of Nestlets on open field locomotion and immediate early gene (IEG) expression. We found that isolation reared rats treated with Nestlets a) healed significantly better than without Nestlets, 2) healed at a similar rate to rats treated with oxytocin, 3) had decreased hyperactivity in the open field test, and 4) had normalized IEG expression in brain hippocampus.

**Conclusions/Significance:**

This study shows that when an EE strategy or oxytocin is given to isolation reared rats, the peripheral stress response, as measured by burn injury healing, is decreased. The findings indicate an association between the effect of nest making on wound healing and administration of the pro-bonding hormone oxytocin. Further elucidation of this animal model should lead to improved understanding of how EE strategies can ameliorate poor wound healing and other symptoms that result from isolation stress.

## Introduction

Recent evidence indicates that environmental enrichment (EE) improves the attachment behavior of rat dams toward their pups, and that this is likely mediated through the effects of EE on the estrogen receptor and its modulation of oxytocin brain levels. [1]. The hippocampus appears to mediate this effect, through changes in the hippocampal glucocorticoid receptor (GR) induced by differences in rat maternal attachment behaviors (licking and grooming) [2]. Changes in the hippocampus resulting from aberrant attachment behaviors alters hypothalamic pituitary axis (HPA) functioning and subsequently has downstream effects on peripheral immunocompetency (as a result of complex changes in the interplay of mineralocorticoid (MR) and GR receptors in the hippocampus) [3]–[7].

Based on the above findings, we hypothesized that 1) EE treatments decrease the peripheral stress response as reflected in poor wound healing, and 2) that the effect of EE on the peripheral stress response is mediated through the central nervous system (CNS) and its effect on the HPA. To test these hypotheses, we examined whether: 1) an EE treatment, which consisted of giving isolation reared rats the opportunity to build nests twice weekly, could reduce the stress response enough to promote wound healing, and 2) whether giving another group of isolation reared rats oxytocin could reduce their stress response and promote wound healing to the same extent as Nestlets.

Of the many potential peripheral stress responses, we chose to look specifically at wound healing, as we had previously shown that rats reared in an impoverished environment (isolation reared rats) had substantially worse wound healing and decreased brain activity in a key region of the brain involved in stress response (the medial prefrontal cortex) [8], [9]. Furthermore, substantial literature indicates that wound healing is impaired by psychological stress. In humans, female caretakers of Alzheimer patients [10], women reporting high levels of general life-stress [11], young adults undergoing an academic exam [12], couples undergoing marital distress [13], and patients with pre-existing psychotic illnesses [14] show delayed wound healing. In rodents, restraint stress impairs wound healing and cytokine expression [15]–[17]. A few studies have shown that interventions in rodents designed to reduce stress can improve wound healing and abnormal behaviors. [18], [19]. Other studies have shown that physical contact facilitates wound healing [20].

We selected nest building as our EE treatment because isolation reared rats are deprived of normal post-weaning bonding, and a key aspect of bonding for rats involves the nest building by the rat pup's dam [21], [22]. Specifically, we examined whether placing Nestlets (Ancare, Bellmore, NY, U.S.) in cages of isolation reared rats could dampen the negative down stream effects of isolation rearing on wound healing. Nest building with Nestlets is associated with anxiolysis [23], hippocampal function [24], reduction of stress hormones [25], and maternal behavior [26].

To evaluate our hypothesis that the mechanism by which the EE of nest building improves wound healing is central (i.e. is due to effects of the Nestlets on the CNS), we gave all isolation reared rats exogenous oxytocin. We then compared the wound healing of animals given Nestlets with those given oxytocin. We reasoned that if the effect of Nestlets on wound healing is centrally mediated through the anxiolytic effects of the Nestlets, then rats treated with oxytocin should have a similar healing response to rats treated with Nestlets. We based this reasoning on the fact that oxytocin, through central mechanisms, enhances social bonding [27], and through its central effect, has a positive impact on the systemic stress response [28]–[30], and on wound healing [18], [19], [31], [32].

In this study we administered oxytocin intraperitoneally. Because centrally delivered oxytocin receptor antagonists block the effects of peripherally delivered oxytocin, it is likely that peripherally delivered oxytocin acts centrally [33]–[35], even though only a small amount of peripherally administered oxytocin crosses the blood brain barrier [34]. In addition, peripherally administered oxytocin alters central adrenergic receptors [36], and brain hippocampal MRs and GRs [37], providing further support that peripherally delivered oxytocin has important central effects.

## Methods

### Animals

The animals were maintained in accordance with National Research Council guidelines and the experimental protocols were approved by the Subcommittee on Research Animal Care, Committee on Research, Massachusetts General Hospital. The study was designed to minimize the number of animals required, and all efforts were made to minimize their suffering. Male Sprague–Dawley rats (Charles River Laboratories, Wilmington, MA, USA and Harlan Sprague–Dawley Inc. Indianapolis, Indiana, USA) were obtained at PN 17 with lactating dams. Rats from all experimental conditions were housed in the same animal room. Rats were killed by rapid decapitation on PN 48 (for experiments 1 and 3) and at PN 62 (experiment 2).

### Experiment 1 – Assessment of Wound Healing Due to Nestlet Treatment

#### Wound Administration

After habituation to the rat facility, on PN20 a 20% dorsal scald burn was performed as follows. PN 20 rat pups were quickly removed from the cage where they resided with their dam. The pups were weighed (rats weighed between 40–50 grams) to determine the volume of anesthesia to be administered. Anesthesia was then rapidly induced with 80 mg/kg ketamine and 12 mg/kg xylazine via intraperitoneal injection. Anesthesia was deemed sufficient when the animal lacked a contracting reflex in response to a toe pinch. Hair surrounding the area to be burned was then removed from the dorsal area using an electric razor. The anesthesia was checked again, and a surface corresponding to 20% of the total body surface area (TBSA) on the animal's back was immersed in water at 92°C for 8s to produce a full-thickness scald injury (prior experiments from this lab and others have determined that a 20% TBSA burn is sufficient to produce major physiological alterations). To accomplish this, the animal was placed, dorsal surface down, in a mold exposing 20% of the skin to preheated water. The mold was constructed with an opening placed at the rats mouth that is high enough to allow continuous breathing without any exposure to water. The rats were immediately resuscitated with saline, 50 ml/kg, via peritoneal injection. At no time were the hindquarters exposed to thermal injury or injections of any kind. Sham control rats were anesthetized, shaven, and resuscitated, but not exposed to thermal injury. Two pups died from the shock of the burn and were thus excluded from the experiment.

#### Wound Healing Rearing Conditions

The rats were divided into 3 different rearing conditions in order to assess the effect of our rat EE (provision of Nestlet enriched cages) on wound healing: a) 4 weeks of group housing (n = 3 per cage), b) 4 weeks of isolation rearing, c) 4 weeks of isolation rearing with Nestlets (Nestlets administration described below). For this experiment we initially examined 22 rats from the Charles River breeding facility (9 group reared rats, 5 isolation reared rats, and 8 isolation reared given Nestlets). To ensure that these results were replicable and because a colleague had found differences in fear conditioning in rats from different breeding facilities, we then examined the same conditions for 9 rats from the Harlan breeding facility (3 group reared, 3 isolation reared, and 3 isolation reared rats given Nestlets). Thus we had a total of 12 burn injured group reared rats, 8 burn injured isolation reared rats, and 11 burn injured isolation reared rats in Experiment 1 for wound healing analysis.

#### Environmental Enrichment Technique (Provision of Nest Building Material to Isolation Reared Rats)

**Figure 1:**
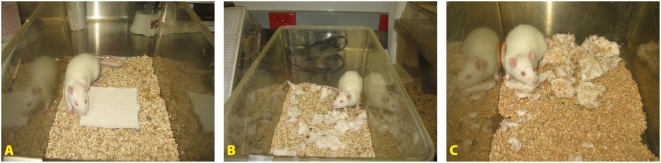
Nestlet “treatment” of isolation reared rats. Twice weekly cages are changed and replaced with a new Nestlet (A), the Nestlet is shredded by the rat prior to forming the nest (B), and then the rat spends time resting in the formed nest (C).

Nestlets are chemically inert, odorless, non-ingestible two-inch squares of sterilized pulped virgin cotton fiber. Twice weekly, EE rats received clean cages with a new Nestlet (Figure 1a), while at the same frequency, rats in the other experimental groups received clean cages without Nestlets. The Nestlet was quickly torn up by the rat (Figure 1b) and over time the rats move the torn pieces toward one side of the cage where they spend time resting (Figure 1c).

#### Assessment of Wound Healing

To assess the healing of the rats in experiment 1, we took pictures of the animals immediately after sacrifice on PN48. The pictures were then analyzed with both a qualitative and quantitative approach. The qualitative analysis was performed as follows: 4 individuals, including 2 experts in wound care (surgical nurse practitioners from Shriners Burns Hospital), rated the wound healing. Inter-rater reliability was 100%. Wounds were rated as “well healing” or “poorly healing”. Rats were qualified as well healing if they met the criterion that a similar type of wound in a patient would be treated conservatively (with a simple dressing), while rats that qualified as poorly healing met the criterion that a similar wound in a patient would require more aggressive treatment than a dressing.

Quantitative assessment of the wound healing was done using the Microsoft Office Picture Manager Program. Using this program, we cropped the picture of each animal to measure the pixels comprising its maximal width. We then cropped the picture further to allow measurement of the pixels comprising the maximal width of each wound's unhealed tissue. The ratio of the unhealed wound tissue to the animal's width served as the quantitative measurement of each animal's wound healing.

### Experiment 2 – Time Series Analysis of Wound Healing and Comparison of Wound Healing with Nestlets to Wound Healing with Oxytocin

#### Wound Injury and Rearing Conditions

Wound injury and wound healing rearing conditions were the same for this experiment as Experiment 1, except that we added a fourth condition, in which isolation reared rats were given oxytocin intraperitoneal injections, as described next.

#### Oxytocin Administration to Rats

Five times weekly for a total of six weeks, rats for each condition (group reared burn injured rats given vehicle (n = 6), isolation reared burn injured rats given vehicle (n = 4), isolation reared burn injured rats given Nestlets and vehicle (n = 6), and isolation reared burn injured rats given oxytocin (n = 6)) were placed under isoflurane anesthesia and administered peritoneal injections of oxytocin (10 mg/kg dissolved in physiological saline, Sigma) or an equal amount of vehicle (saline). The dose of oxytocin was based on previous studies in which peripherally administered oxytocin had wound healing or central effects [18], [32], [35]–[37].

#### Assessment of Wound Healing

To assess the healing of the rats in experiment 2, we examined pictures of the animals taken under anesthesia while they were receiving either vehicle or oxytocin injections as described above. The pictures were then analyzed as follows. Each picture was taken from approximately the same height. A small ruler was placed next to the rat prior to the picture was taken. The unhealed tissue for each rat was outlined using Metamorph software (http://www.moleculardevices.com/pages/software/metamorph.html). The number of pixels comprising this area was then normalized against the pixels comprising one square inch of the ruler in the picture and against the area of the animal (determined by multiplying head to tail distance by greatest width of the body). Normalizing to the ruler eliminated variations in wound size that owed to differences in the distance of the camera to the animal, and normalization to the area of the animal accounted for differences in wound size measurement that owed to the size of the animal (a bigger animal might have a larger area of unhealed tissue but that did not comprise a larger TBSA of unhealed tissue).

### Experiment 3 – Assessment of Behavior and Brain Changes due to Nestlet Administration

#### Rearing Conditions

For these studies, rearing conditions were the same as for the experiments above with two exceptions. First, no burn injury was applied to these animals, as we wanted to assess the effect of Nestlets on brain and behavior without the confounding effects of the burns. In a prior study, we found the burn injury did alter both brain and behavior function [8]. A study now underway in our laboratory is examining brain and behavior changes due to Nestlets after a burn injury. Second, we added a fourth group to this experiment: application of Nestlets to group reared rats (we did not have this condition in the wound healing studies above, since there could be no significant further contribution of Nestlets to the group reared rats' almost complete healing).

#### Assessment of Nestlet Treatment on Locomotion

Based on the above reasoning, we tested locomotion in the open field for the following experimental groups: a) group reared with no burn (n = 12), b) group reared with no burn but with Nestlet treatment (n = 12), isolation reared with no burn (n = 10), and isolation reared with no burn but with Nestlet treatment (n = 11). On PN 38 (after 18 days of group or isolation rearing with or without Nestlets), the rats in each condition were tested in locomotor boxes for behavior in the open field (Med Associates, St. Albans, VT, USA). All testing was carried out over five consecutive hours on a single day. More details of the open field testing procedure, including how movement is measured, are provided in our previous publications [8], [9].

#### Assessment of Nestlet Treatment on Gene Expression

The rats tested in the open field test were sacrificed by rapid decapitation on PN48 (7 days after the open field test so that we were measuring basal levels of gene expression, not the effect of the open field test). After sacrifice, whole brains were immediately frozen in 2-methylbutane and stored at −80°C. The areas of the brain comprising the hippocampus (lateral oriens, pyramidal cell, lateral hippocampus, stratum lucidem, stratum radium, granular and polymorph lateral dentate gyrus; from bregma AP: −0.29 to −2.30) and medial prefrontal cortex (prelimbic cortex and medial orbital cortex; from bregma AP: +3.08 to +1.54) [38] were dissected on a freezing microtome.

RNA was extracted from approximately 20–30 mg of tissue using the Invitrogen total RNA extraction kit (www.invitrogen.com). Total RNA quality was assessed by spectroscopy, and where deemed adequate, was reverse transcribed to cDNA using the Two Step RT-PCR Kit (Invitrogen) following the manufacturer's instructions in a Perkin Etus Thermal Cycler 480.

The gene expression patterns were assessed using quantitative PCR (qPCR). cDNA was analyzed by qPCR using the Stratagene mx3005P instrument (www.stratagene.com) with the following cycling conditions: step 1) 55°C for 2 min and 95°C for 2 min; step 2) amplification at 95°C for 30 sec, 58°C for 30 sec, and 72°C for 50 cycles. A melting curve was used to confirm the specificity of each primer pair. Each sample was run in triplicate to exclude outliers.

Primers used for amplification were designed using Primer3 (www-genome.wi.mit.edu/cgi-bin/primer/primer3.cgi.) for amplicons between 100 and 200 base pairs (see Table 1 for primer sequences).

**Table 1:** Entrez Gene ID Numbers and Primer Sequences of Genes Used for Quantitative Polymerase Chain Reaction Experiments

Gene	Entrez Gene No.	Forward Sequence	Reverse Sequence
Arc/Arg3.1	23237	GGT GTC ATT CAC CTG GCT CT	AGT CTT GGG CAG CAT AGC TC
cFos	2353	GAA GGA ACC AGA CAG GTC CA	TCA CCC TGC CTC TTC TCA AT
Junb	3726	TAT GGA GCA AGG GAG GCT CT	CCT GGA GGA CAA GGT GAA GA
NGFI-B	3164	TCC AGC TTG AGG CAA AAG AT	TGC TCT GGT CCT CAT CAC TG
β-actin	450133	GTC GTA CCA CTG GCA TTG TG	TCT CAG CTG TGG TGG TGA AG

Gene expression was analyzed using the ΔΔCT method, using β-actin as the normalizer gene. After elimination of outliers (the criterion for an outlier was a ΔΔCT value greater or lower than one SD from the mean of the rats in a particular condition) and tissue with inadequate RNA quality based on spectroscopy analysis, we computed the average gene expression for each experimental condition (group reared rats with Nestlets, n = 6; isolation reared rats with Nestlets, n = 4; and isolation reared without Nestlets, n = 8) relative to the control condition (group reared rats without Nestlets, n = 12).

## Results

### Experiment 1 – Effect of Nestlet Treatment on Wound Healing

**Figure 2:**
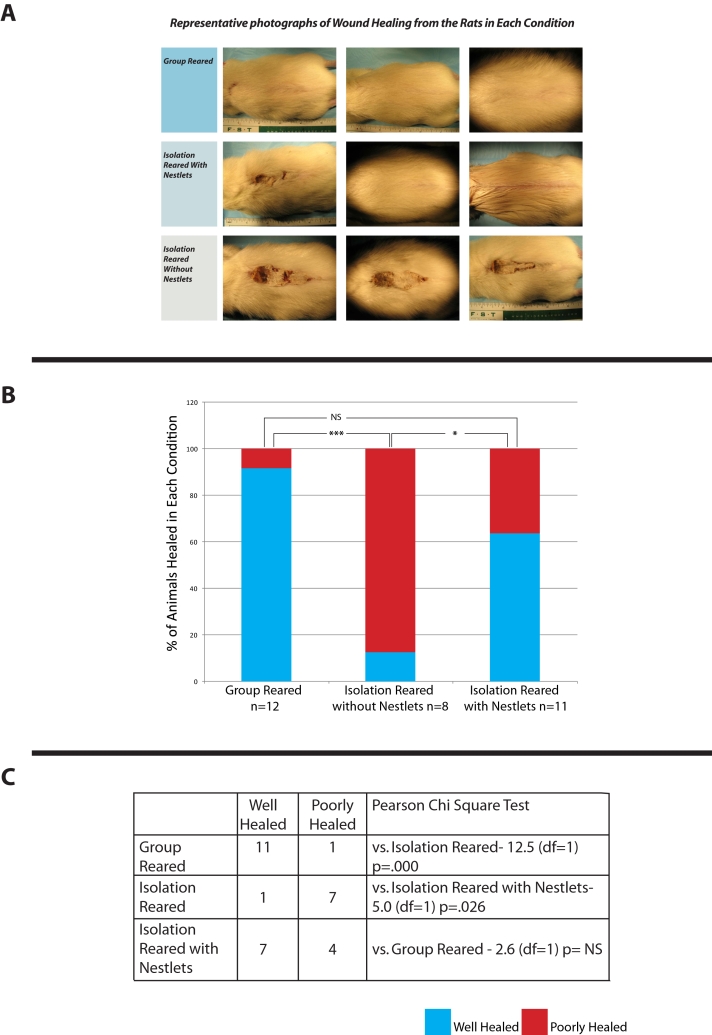
Example of healing in rats in the different conditions examined (A). 92% of group reared rats healed well (n = 12, column 1, 2B, and top row 2C), 12% of isolation reared rats healed well (n = 8, middle column 2B, and middle row 2C), and 64% of isolation reared rats treated with Nestlets healed well (n = 11 see third column 2B, and bottom row 2C).* *P*<0.05, ** *P*<0.01, *** *P*<0.001.

Wound healing at PN 48 in the 31 Charles River and Harlan bred rats (10 group reared, 8 isolation reared, and 11 isolation reared with Nestlets) was assessed in this experiment. Figure 2A shows examples of healing in rats from each of the rearing conditions. We found that 92% of the group reared rats met the criterion described in the Methods Section for well healed, while only 12% of the isolation reared rats without the Nestlets met this criterion (Figure 2B). On the other hand, 64% of isolation reared rats who received the Nestlets were considered well healed (Figure 2B). The chi square test showed the difference between group reared rats and isolation reared rats without Nestlets to be significant, while the difference between the group reared and isolation reared rats with the Nestlets was not significant (Figure 2C).

**Figure 3:**
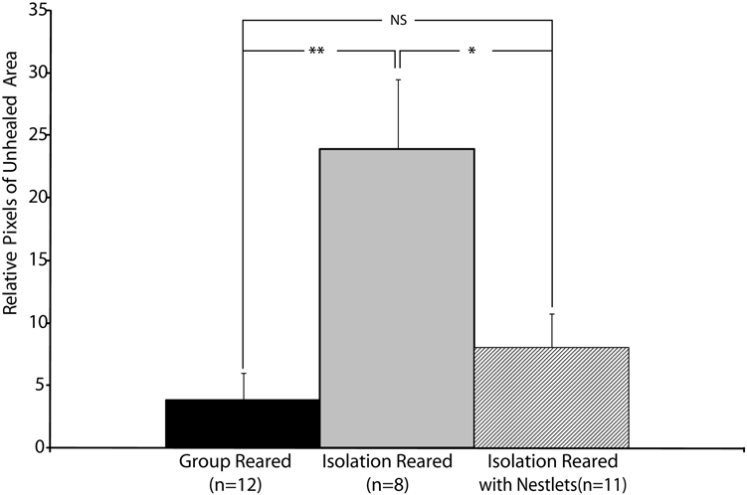
Degree of impaired burn healing rats in each condition. For each rat, the number of pixels comprising the width of the maximum gap of unhealed tissue was normalized to the width of its back. The average normalized pixels of unhealed tissue were significantly greater for the isolation-reared rats (middle column) compared with both the group-reared rats (first column) and the isolation reared rats treated with Nestlets (third column). Average±S.E.M., *p<.05, ** *P*<0.01.

Quantitative analysis of the unhealed wound area for rats in each category (combining data from both the experiments with the Charles River and the Harlan rats) showed significantly greater wound healing in both group reared and isolation reared rats that received Nestlets compared to isolation reared rats that did not receive Nestlets (Figure 3).

### Experiment 2 – Time Series Analysis of Wound Healing and Effect of Oxytocin on Wound Healing

The purpose of this experiment was three fold: a) to obtain a more precise quantification of the wound healing in the different conditions, b) to determine whether the wounds healed at a different rate in the different rearing conditions, and c) to determine if oxytocin, a pro-bonding (affiliation enhancing) hormone, administered to the rats would improve healing to a similar degree as the Nestlets when administered to the rats.

**Figure 4:**
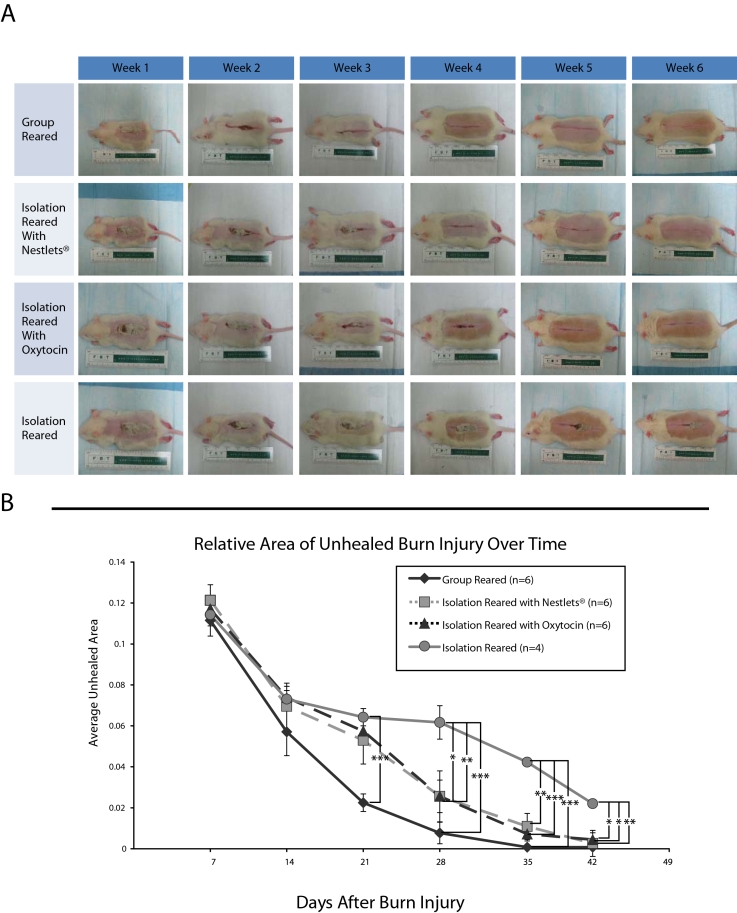
Time series analysis of Nest Building and Oxytocin effect on wound healing. Panel A shows an example of wound healing over six weeks from weaning (PN20 to PN62) in the four conditions examined. Panel B shows that group reared rats had significantly better healing compared to isolation reared rats by 21 days post burn injury, while Nestlet and oxytocin treated rats showed similar healing to group reared rats by 28 days post burn injury. The difference between Nestlet treated, oxytocin treated, and group reared rats compared to isolation reared rats continued until 42 days post burn injury. Average±S.E.M., *p<.05, ** p<0.01, ***p<0.001.

As detailed in the Methods Section, using Metamorph (http://www.moleculardevices.com/pages/software/metamorph.html), we measured the size of the unhealed tissue in each animal more precisely for this experiment than for Experiment 1. With this approach, we found that, as shown in Figure 4, the group reared rats healed superior to the isolation reared rats by 21 days after the burn injury (PN41). By 28 days after the burn injury (PN48) both treatment with Nestlets and oxytocin resulted in superior healing to the isolation reared rats. The superior healing of rats: a) reared in groups, b) reared with Nestlets, and c) reared with oxytocin injections relative to isolation reared rats continued through 42 days post burn injury (PN62). Of note, as shown at the last time point in Figure 4, the healing of the isolation reared rats began to approach that of the other conditions at the time of sacrifice (42 days post weaning; PN62), but remained statistically different.

Overall the results of this experiment show 3 main findings: 1) that treatment with oxytocin approximates that with Nestlets, 2) the rate of improvement is similar for the rats treated with Nestlets and oxytocin, and is slower than group reared rats, but faster than the isolation reared rats, and 3) that the isolation reared rats heal at a much slower rate with a still significantly greater area of unhealed tissue compared to rats in the other rearing conditions at 42 days post burn (two weeks beyond the point when wound healing was assessed in experiment 1).

### Experiment 3 – Effect of Treatment with Nestlets on Behavior and Brain

We hypothesized that Nestlets had their effect on wound healing by affecting the central nervous system. Some support for this was obtained in Experiment 2, which showed that the affiliative hormone oxytocin improved wound healing at a similar rate to that of the Nestlets. To further examine whether the Nestlets positively affected wound healing through a central mechanism we examined whether behaviors and brain changes associated with isolation rearing were reversed by Nestlets.

#### Behavioral Effect of Nestlet Treatment

**Figure 5:**
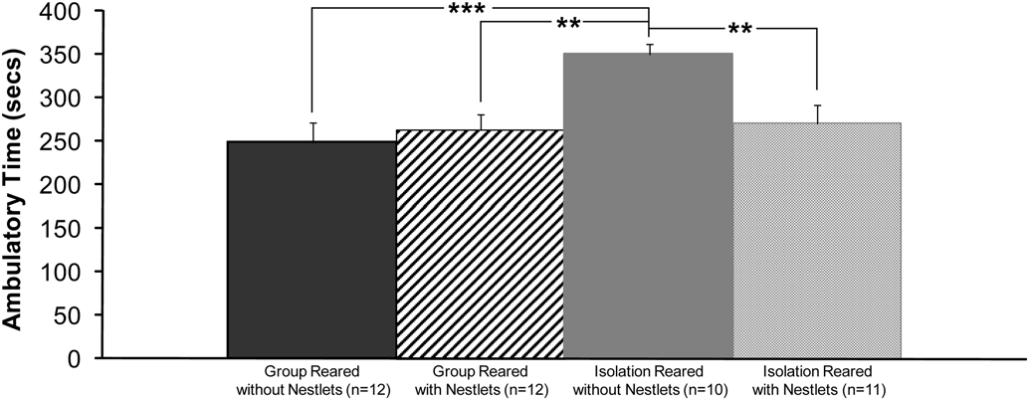
Effect of Nestlet treatment on open field test behavior. Ambulatory time was significantly lower for isolation reared rats treated with Nestlets (column 4) compared to untreated isolation reared rats (column 3) and not different from group reared rats (column 1) or group reared rats treated with Nestlets (column 2). Average±SEM.,*P<0.05, ** *P*<0.01, ***P<0.001.

Open field hyperactivity that we and others have previously shown to be present in isolation reared rats compared to group rats [8], [9], [39] was absent in Nestlet-treated isolation reared rats as shown in Figure 5.

#### Hippocampus and Medial Prefrontal Cortex (mPFC) Gene Expression Changes with Nestlet Treatment

**Figure 6:**
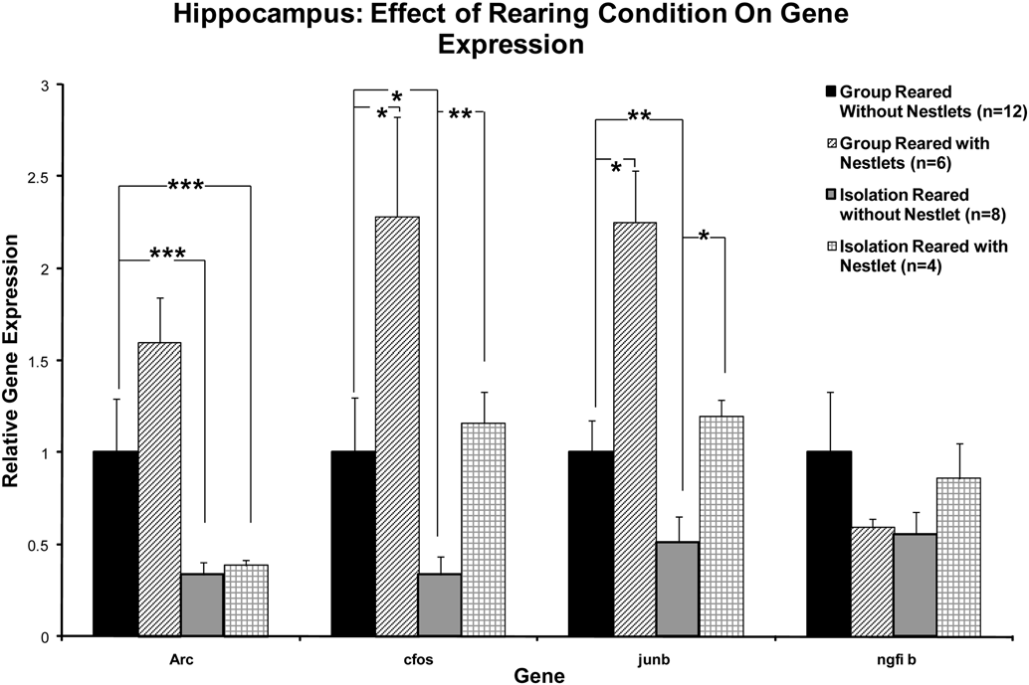
Gene expression changes in the hippocampus by condition. Rats treated with Nestlets had significantly higher gene expression compared to isolation reared rats without Nestlets for cfos and junb (compare columns 3 and 4 for these genes). Gene expression of rats treated with Nestlets returned to that of group reared rats for these genes (compare columns 1 and 4 for cfos and junb). Group reared rats treated with Nestlets showed an increase in these genes above the expression level for group reared rats not treated with Nestlets (compare columns 1 and 2) even though there was no additional benefit to their wound healing (since wound healing was maximal for the group reared rats without the Nestlets). Average±SEM, *p<0.05, **p<.01, ***p<.001.

To determine if the improvements in wound healing and reduced hyperactivity resulting from Nestlet treatment were associated with changes in neural activity, we examined gene expression in the hippocampus and mPFC, regions having been established as key to the stress response [40], [41]. We examined four immediate early genes (IEGs) that we had previously shown to be altered in isolation reared rats with poor wound healing [8]. In the hippocampus, two of the four IEGs (cFos and Junb) had significantly increased gene expression in the isolation reared rats treated with Nestlets compared to isolation reared rats without Nestlet treatment (compare columns 3 and 4, Figure 6, for these 2 genes). Furthermore, for these two genes, the gene expression of isolation reared rats treated with Nestlets returned to that of group reared rats in the hippocampus (as shown by the lack of statistical difference between columns 1 and 4, Figure 4, for these two genes). The same two genes (cFos and Junb) showed a non-significant trend toward increased expression (p = .08 and .09, respectively) in the mPFC of isolation reared rats treated with Nestlets compared to isolation reared rats without Nestlets,

## Discussion

This study examined two key questions, 1) whether providing environmental enrichment (EE), specifically nest building opportunities, to rats would result in a tangible change in their physical health, and 2) whether this effect involves the central nervous system.

### The Effect of EE on Physical Health

As we had previously shown that an impoverished environment impaired wound healing, we focused on wound healing as our measure of physical health. We found strong support for our hypothesis in that nest building almost completely resolved the impaired wound healing that resulted from isolation rearing. To the best of our knowledge, this is the first time that a non-pharmacological strategy has been demonstrated to treat impaired healing of third degree burns. The results are certainly consistent with earlier work suggesting that social EE can reverse the negative effects of isolation rearing, possibly through an oxytocin mediated mechanism [1]. However, in this study, the EE was not social, although nest making is associated with maternal behavior [26].

Further insight into the effect of EE on wound healing was obtained in experiment 2 where we observed that the rate of wound healing was different for the group reared rats compared to the rats treated with Nestlets or oxytocin. Although, by 28 days post burn injury (PN48), the healing was similar among all three of these groups compared to the isolation reared rats, the group reared rats' healing was substantially improved by 21 days post burn injury, while the Nestlet and oxytocin treated rats did not substantially improve until 28 days post burn injury relative to the isolation reared rats. This suggests that the Nestlet treatment alters the rate of the healing response in addition to its cumulative effect on healing. Even among the untreated isolation reared rats, there was some evidence that healing began to occur by the time of sacrifice (42 days post burn injury), but the degree of unhealed tissue, even at this date, was still significantly greater than the other experimental conditions (group reared, Nestlet treated and oxytocin treated rats). Thus, it may be that these interventions affect the speed of the healing response in addition to having an overall net effect on wound healing. The neuroendocrinological and neuroimmunological mechanisms by which this EE treatment resulted in a more expeditious peripheral healing process are important targets of future research.

### The Role of the Central Nervous System in the EE Treatment

The mechanism by which this EE strategy operates to repair wounds in isolation reared rats is still unclear. As stated in the introduction, our working hypothesis is that the mechanism by which our EE intervention improves wound healing involves the central nervous system, and its downstream effects on the peripheral healing. While the evidence from this study does not allow us to draw conclusions about the mechanism by which our EE intervention improved wound healing, we did obtain evidence to indicate that this EE intervention impacts the central nervous system. First, hippocampal expression of immediate early genes, a measure of brain neural activity [42], [43], increased in isolation reared rats given Nestlets compared to isolation rearing without Nestlets. This provides evidence that the hippocampus is a brain region that the Nestlets either directly or indirectly target. Second, isolation reared rats with Nestlets evidenced reduced hyperactivity in the open field test, a behavior that is likely mediated, in part, through the hippocampus as open field hyperactivity is thought to result from deficient habituation to a novel environment [44] and habituation to novelty likely involves the hippocampus [45]. Finally, we found that delivering the pro-bonding hormone oxytocin improved wound healing among isolation reared rats at the same rate as the isolation reared rats provided with Nestlets. This hormone has numerous effects on the brain, including quantitative changes in hippocampal GRs and MRs [37], enhancement of social bonding [27], and altered central adrenergic receptor density [36].

The finding that the hippocampus rather than the mPFC showed the most robust IEG expression changes with Nestlet treatment is consistent with the finding that nest building appears to reflect brain hippocampus function [24]. Of interest, the gene with the greatest brain expression change in our prior studies [8], [9] by isolation rearing (Arc) was not affected by Nestlet treatment. Burrowing, a behavior related to nest building was impaired in rats with a potassium channel defect [46], a different excitatory mechanism for cells. We can speculate that nest building effects might be mediated through an alternate pathway such as the potassium channel, rather than a glutamate channel (that Arc modifies).

### Study Limits, Future Directions, Conclusions

At present, our findings only indicate a causal link between our EE treatment, as well as oxytocin, and improved wound healing in isolation reared rats. The findings with regard to the brain changes induced by Nestlets establish that this EE treatment is associated with both brain and wound healing changes. However, these findings do not establish a causal link between these brain changes and the wound healing. Whether these two effects of the EE treatment are linked mechanistically will require further study. We have started to examine this question in our laboratory in a study that delivers a central oxytocin receptor antagonist and observing whether it blocks the beneficial effect of both treatment with Nestlets and oxytocin on wound healing. Furthermore, in this study we are examining peripheral stress hormone levels to see if these are altered by treatment with Nestlets, oxytocin, and oxytocin receptor antagonists.

Also, while we can conclude that oxytocin mimicked the beneficial effect of nest building on impaired wound healing in isolation reared rats, we cannot be certain that the wound healing changes resulting from provision of Nestlets owes to the same mechanism as the wound healing that resulted from the oxytocin, as oxytocin has both central and peripheral mechanisms [27]–[29], [31], [33], [34], [36], [37], [47]. Our current study described above should provide significant insight into whether oxytocin alters wound healing through a similar pathway to that of the Nestlets.

Nonetheless, this study clearly establishes that brain, behavior, and wound healing are all altered by both the EE of nest building and oxytocin. In total, the findings indicate an association between the effects of nest making on wound healing in isolation reared rats and administration of the pro-bonding hormone oxytocin. Thus, this animal model can potentially be exploited in future studies to develop behavioral and pharmacological strategies to treat impaired physical health that has a central or “stress” based component, particularly stress due to social isolation, neglect, or deprivation states.
